# Vestibular Endolymphatic Hydrops Visualized by Magnetic Resonance Imaging and Its Correlation With Vestibular Functional Test in Patients With Unilateral Meniere's Disease

**DOI:** 10.3389/fsurg.2021.673811

**Published:** 2021-06-04

**Authors:** Yupeng Liu, Fan Zhang, Baihui He, Jingchun He, Qing Zhang, Jun Yang, Maoli Duan

**Affiliations:** ^1^Department of Otorhinolaryngology-Head & Neck Surgery, Xinhua Hospital, Shanghai Jiaotong University School of Medicine, Shanghai, China; ^2^Ear Institute, Shanghai Jiaotong University School of Medicine, Shanghai, China; ^3^Shanghai Key Laboratory of Translational Medicine on Ear and Nose Diseases, Shanghai, China; ^4^Ear Nose and Throat Patient Area, Trauma and Reparative Medicine Theme, Karolinska University Hospital, Stockholm, Sweden; ^5^Division of Ear, Nose and Throat Diseases, Department of Clinical Science, Intervention and Technology, Karolinska Institutet, Stockholm, Sweden

**Keywords:** Meniere's disease, vestibular function, MRI, endolymphatic hydrops, VEMP, vHIT, caloric test

## Abstract

**Background:** Currently, 3 Tesla-MRI following intratympanic gadolinium injection has made it possible to assess the existence and the severity of hydrops in each compartment of the endolymphatic spaces *in vivo*. However, the relationship between vestibular endolymphatic hydrops (EH) visualized by MRI and vestibular functional tests, especially the correlation between caloric test, video-head impulse test, and semicircular canal hydrops, has not been well-investigated.

**Objective:** The purpose of this study is to investigate the relationship between the severity of EH in each compartment of otoliths and semicircular canal and the results of vestibular functional tests.

**Methods:** In this retrospective study, we performed three-dimensional fluid-attenuated inversion recovery (3D-FLAIR) sequences following intratympanic gadolinium injection in 69 unilateral patients with definite Menière's disease. Vestibular and lateral semicircular canal hydrops was graded on MRI using a four grade criterion. All patients underwent pure-tone audiometry, cervical vestibular evoked myogenic potential (cVEMP), ocular vestibular evoked myogenic potential (oVEMP), caloric test and video head impulse test (vHIT). The latency, amplitude and asymmetry ratio of VEMP, canal paresis (CP) and vestibulo-ocular reflex (VOR) gain of lateral semicircular canal of vHIT were collected. The correlation analysis were performed between the parameters of function test and EH.

**Results:** Vestibular EH showed correlations with the duration of disease (*r* = 0.360) and pure tone average (*r* = 0.326). AR of cVEMP showed correlations with Vestibular EH (*r* = 0.407). CP (*r* = 0.367) and VOR gain of lateral semicircular canal at 60 ms (*r* = 0.311) showed correlations with lateral semicircular canal hydrops.

**Conclusion:** EH in different compartments is readily visualized by using 3D-FLAIR MRI techniques. The degree of vestibular EH correlated with AR of cVEMP and EH in the semicircular canal ampullar affects the caloric and vHIT response in patients with unilateral Meniere‘s disease.

## Introduction

Ménière's disease (MD) is an idiopathic inner ear, which is characterized by recurrent attacks of vertigo, fluctuating hearing loss, tinnitus, and ear fullness. The Etiology of the disease is unclear and the pathological feature is endolymphatic hydrops (EH) (1). At present, a clinical diagnosis of MD is mainly based on the combination of medical history and auditory-vestibular function examination. With the combined application of different vestibular function examinations, clinicians can evaluate the function of saccule, the utricle, the low frequency of semicircular canal and the high frequency of semicircular canal separately by cervical vestibular-evoked myogenic potentials (cVEMP), ocular vestibular-evoked myogenic potentials (oVEMP), Caloric test, and video-head impulse test (vHIT) (2–4). Since 2007, the morphological assessment of EH in MD patients on magnetic resonance imaging (MRI) has been realized by the application of 3-dimensional fluid-attenuated inversion recovery (3D-FLAIR) sequence following the intratympanic injection of gadolinium (Gd) by Nakashima et al. (5). With the development of imaging technology, various structures of the inner ear have been observed by intratympanic or intravenous injection of contrast media.

In previous studies, many authors have reported the correlation between the degree of vestibular hydrops and vestibular function examination results in MD patients. But the conclusion is inconsistent. Guo et al. (6) reported that the asymmetry ratio (AR) of oVEMP showed correlation with the severity of vestibular EH. Katayama et al. (7) confirmed that EH was significantly associated with the disappearance of cVEMP. On the contrary, Kahn et al. (8) concluded that no significant correlation between the presence of EH and vestibular function was found in the research. The correlation between the caloric test and EH is also inconclusive. Kato et al. (9) presented that there was no significant correlation between the caloric test results and the degree of EH in the vestibule, or the lateral semicircular canal ampulla. While, Sluydts et al. (10) and Guo et al. (6) reported that the abnormal caloric tests was correlated with the severity of vestibular. Moreover, few studies have focused on the relationships between semicircular canal hydrops and vestibular-ocular reflex (VOR) recorded by video head impulse test (vHIT) (11, 12).

The purpose of this study is to elucidate the relationships between the results from cVEMP, oVEMP, the caloric test, vHIT, and EH visualized by Gd-enhanced MRI in patients with unilateral MD.

## Materials and Methods

### Patients

This study was approved by the Ethics Committee of Xinhua Hospital, Shanghai Jiaotong University School of Medicine. Written informed consent was obtained from each participant. We retrospectively reviewed the medical records of 69 patients diagnosed with definite unilateral MD who were admitted to Department of Otolaryngology-Head and Neck Surgery, Xinhua Hospital, Shanghai Jiaotong University School of Medicine from March, 2018 to December, 2020. The diagnostic criteria was based on the 2015 consensus of the Barany Society (13). The inclusion criteria were as follows: (1) unilateral MD patients without complains, such as tinnitus, ear fullness, or hearing loss in the contralateral ears; (2) complete medical history record with auditory-vestibular function examinations and MRI. The exclusion criteria were as follows: (1) chronic otitis media or other middle or inner ear disease history; (2) history of middle or inner ear surgery; (3) receipt of intratympanic gentamicin or dexamethasone treatment; (4) use of vasodilators or diuretics in the previous 2 weeks; (5) patients with claustrophobia, pregnancy, or Gd allergy.

### Intratympanic Gd Injection and MRI

As our previous studies described, the Gd contrast medium (Omniscan, Xudonghaipu Pharmaceutical Co. Ltd, Shanghai, China) was diluted 8-fold with saline (v/v, 1: 7). Approximately 0.4–0.6 ml of the diluted Gd was injected intratympanically. The injection was performed under a microscope. The patient then was placed in the supine position for 60 min (14).

Twenty-four hours after the Gd injection, MRI scans were performed with a 3.0 Tesla MR scanner (UMR 770, united-imaging, Shanghai, China), using a 24-channel head coil. 3D-FLAIR imaging was subsequently performed. The scan parameters for the 3D-FLAIR sequence were as follows: time of repetition = 6,500 ms, time of echo = 286.1 ms, time of inversion = 1,950 ms, flip angle = 67°, slice thickness = 0.6 mm, echo train length = 160, field of view = 200 ^*^ 200 mm, and matrix size = 256 ^*^ 256, voxel size = 0.78 ^*^ 0.78 ^*^ 1.1 mm. The scan time was 6 min 11 s. Three dimensional heavily T2-weighted spectral attenuated inversion recovery (3D-T2-SPAIR) sequence was also performed with the following parameters: voxel size = 0.65 ^*^ 0.52 ^*^ 0.76 mm, scan time = 4 min 30 s, TR = 1,300 ms, TE = 254.7 ms, flip angle = 110°, slice thickness = 0.4 mm, field of view = 200 ^*^ 200 mm, and matrix size = 384 ^*^ 384 (15).

### Image Analysis

**Figure 1:**
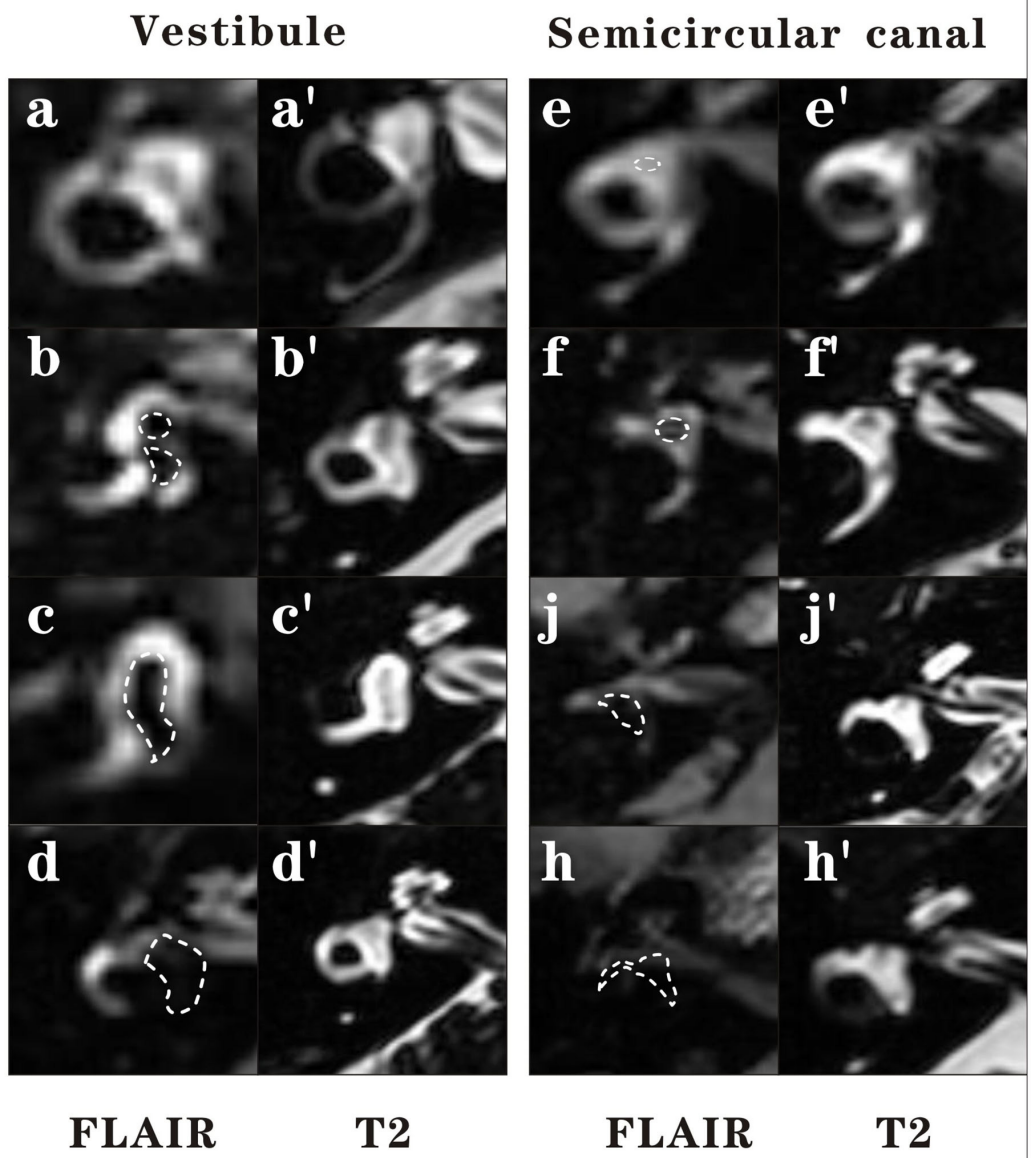
Four grades of the vestibular EH and lateral semicircular canal EH. In this figure, 3D-FLAIR pictures **(a–h)** are shown adjacent to 3D-T2-SPAIR pictures **(a′-h′)** for each grade. For vestibule, grade 0 = No hydrops **(a)**. The saccule and utricle are visibly separately and take less than half of the surface of the vestibule. Grade 1 = Mild hydrops **(b)**. When the saccule become equal or larger than the utricle, but is not yet confluent with the utricle. Grade 2 = Moderate hydrops **(c)**. There is a confluence of the saccule and utricle with still a peripheral rim enhancement of the perilymphatic space. Grade 3 = Severe hydrops **(d)**. The perilymphatic enhancement is invisible. For lateral semicircular canal, grade 0 = No hydrops **(e)**. A small visible herniation which is 1/3 less than the semicircular canal with perilymph surrounding can be observed. Grade 1 = Mild hydrops **(f)**. A dark area occupying over 1/3 of the ampulla is observed. Grade 2 = Moderate hydrops **(g)**. The crura become dark and some of the canals become invisible with hydrops, which often accompany the stenosis of canals. Grade 3 = Severe hydrops **(h)**. All semicircular canals were invisible.

The images were evaluated by an experienced neuroradiologist who was blinded to the patient's information. The vestibule and semicircular canal were separately evaluated. The vestibular part was evaluated using the semi-quantification four-grade system proposed by Bernaerts et al. (16). In brief, a normal vestibule is described as when the saccule and utricle are visibly separately and take less than half of the surface of the vestibule. When the saccule become equal or larger than the utricle, but is not yet confluent with the utricle, vestibular hydrops grade I is defined. When there is a confluence of the saccule and utricle with still a peripheral rim enhancement of the perilymphatic space, vestibular hydrops grade II is defined. When the perilymphatic enhancement is invisible, vestibular hydrops grade III is defined. The lateral semicircular canal was evaluated by the four-grade system proposed by our team in the previous study (15). We defined Grade 0 as no hydrops when a small visible herniation which was 1/3 less than the semicircular canal with perilymph surrounding, a larger herniation (>1/3) with hydrops as Grade I and the total invisibility of crura, which often accompany the stenosis of canals as Grade II. If all semicircular canals were invisible, we defined as Grade III. Images of each grade of vestibular and lateral semicircular canal were demonstrated in Figure 1.

### Audio-Vestibular Functional Tests

#### Audiometry

Pure-tone audiometry was performed in a soundproof room with the use of an audiometer (Type Madsen, Astera, Denmark). The pure-tone average (PTA) is the average of the 0.5, 1, and 2 kHz air conduction thresholds. The calculation is based on the MD guideline of China published in 2017 (17).

#### VEMP

VEMP tests were performed with a Bio-logic Navigator PRO auditory brainstem response diagnosis system. For cVEMP, electrodes were placed on the middle of each sternocleidomastoid muscle. The reference electrode was placed on the middle of clavicular joint and the ground electrode was placed on the nasion. The resistance between the electrodes was controlled at <5 kΩ. Tone bursts (95 dB normal hearing level [nHL], 500 Hz, rise/fall time1 ms, plateau time 2 ms, stimulation frequency 5 Hz, superposition 50 times) were delivered through headphones. Patients are instructed to raise their head from the pillow and supine head 30° after hearing a single stimulus to maintain sternocleidomastoid muscle in tension until the stimulus stops. A well-repeated wave form with p13 and n23 was defined as “Elicited.” Absence of a meaningful wave form with p13 and n23 was defined as no response. The P13 potential was identified as the first positive peak in the waveform, N23 was identified as the first negative peak in the waveform, and the peak-to-peak amplitude was the P13–N23 amplitude. The latency of P13 and N23, the amplitude of P13 and N23, and the amplitude of the first positive–negative peak (P13–N23) was recorded. The percent VEMP asymmetry ratio (AR) was calculated with the following formula using the amplitude of p13–n23 on the affected side (Aa) and that on the unaffected side (Au): VEMP asymmetry (%) = 100 (Au – Aa)/(Au + Aa) %. When only one side of the oVEMP was absent, the AR was calculated as 100%. A value of AR > 40% was considered abnormal (4).

For oVEMP, the setting parameters are the same as above. The electrode was placed 1 cm below the center of the opposite eyelid. The reference electrode was placed in the lower jaw and the ground electrode was placed on the nasion. Ask the patient to gaze upward after hearing a single stimulus. Keep the eye in 25–30° angle and blink as little as possible to maintain the tension of the inferior oblique muscle until the stimulus stops. A value of AR > 40 was considered abnormal (11).

#### vHIT

vHIT was performed with a video-oculography device (Interacoustics, EyeSeeCam, Denmark). The subject was instructed to maintain the fixation on an earth-fixed target, which is usually straight ahead. Experienced otologists An experienced laboratory technician delivered at least 20 brief, abrupt, and unpredictable head impulses per side (10–20° angle, duration 150–200 ms, peak velocity of >150°/s). The VOR gain was defined as the ratio of the eye velocity (°/s) over the head velocity (°/s). Individual VOR gains were automatically calculated using the device software. vHIT testing was considered to be abnormal for horizontal canal if VOR gain at 60 ms was <0.8 (18).

#### Caloric Test

The patients were placed in supine position by raising their head 30° to keep the horizontal canal in a vertical position. The external auditory canal was irrigated with 24°C cold air for cold testing and 50°C hot air for hot testing. The induced nystagmus was recorded using electronystagmography in a dark, open-eye situation. The percentage of canal paresis (CP%) was calculated using Jongkees' formula (19). A CP% of >25% was defined as caloric weakness.

### Data Analysis

All data were analyzed with SPSS v. 25 statistical software (IBM Corp., NY, US). Continuous data are presented as mean with standard deviation. The Wilcoxon rank sum test was used for non-normal data. The chi-square test was applied to compare dichotomous variables among the groups. The Spearman correlation analysis was used for categorical data to explore the correlation between the severity of hydrops on MRI and audiovestibular tests. *P*-values < 0.05 were considered significant.

## Results

### Demographics

The study population consisted of 34 men and 35 women (40 right-sided, 29 left-sided), with ages ranging from 28 to 75 years (mean age 52.62 ± 12.11 years). The mean duration of disease was 3.43 ± 2.86 years. The mean PTA was 55.92 ± 20.56. Based on the level of hearing loss, 5 (7.2%) patients met the criteria for Stage I. Nine (13.0%) patients could be classified as stage II, whereas Stage III and Stage IV comprised 41 (57.9%) and 14 (20.3%) of the patients, respectively. The baseline characteristics of the patient population are summarized in Table 1.

**Table 1:** Characteristics of the study population.

**Characteristics**	***N* = 69**
Age (years)	52.62 ± 12.11
**Sex**	
Male	34 (49.3%)
Female	35 (50.7%)
**Affected side**	
Left	29 (44.6%)
Right	40 (55.4%)
Duration of disease (years)	3.43 ± 2.86
Mean PTA (dB)	55.92 ± 20.56
**Stage**	
I	5 (7.2%)
II	9 (13.0%)
III	41 (57.9%)
IV	14 (20.3%)

*Data are presented as mean ± standard deviation or number (%).*

*Staging was based on the pure-tone average at 0.5, 1, and 2 kHz*.

### MRI Data

**Figure 2:**
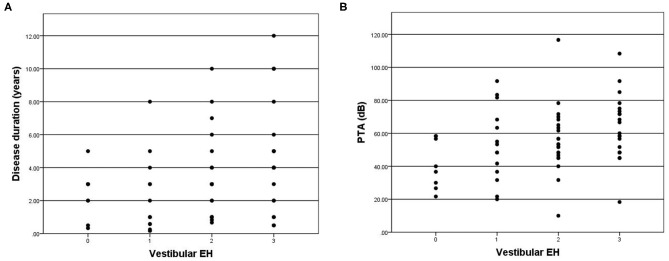
**(A)** Correlation between grade of vestibular EH (x-axis) and disease duration (y-axis). The positive correlation is significant (*r* = 0.360, *p* = 0.003). **(B)** Correlation between grade of vestibular EH (x-axis) and PTA (y-axis). The positive correlation is significant (*r* = 0.326, *p* = 0.008).

Four patients were excluded in the final data analysis because the contrast medium could not even be observed in the any part of membrane labyrinth. Poor permeability of the round window membrane is the most probable reason. Variable degrees of vestibular hydrops were observed in 57 of 65 (87.7%) patients in the affected ear (Table 2). According to the vestibular hydrops grade system, 8 (12.3%) were in grade 0, 14 (21.5%) were in grade 1, 22 (33.8%) were in grade 2, and 21 (32.3%) were in grade 3. For the ampulla hydrops of lateral semicircular canal part, 29 (44.6%) were in grade 0, 23 (35.4%) were in grade 1, 8 (12.3%) were in grade 2, and 5 (7.7%) were in grade 3. A total of three patients had normal vestibule and semicircular canal on MRI. The severity of vestibular hydrops showed correlations with the duration of disease (*r* = 0.360, *p* = 0.003) (Figure 2A) and PTA (*r* = 0.326, *p* = 0.008) (Figure 2B). Vestibular hydrops is not correlated with age of patients (*p* = 0.634). No correlations were observed between lateral semicircular canal hydrops and duration of disease (*p* = 0.582), PTA (*p* = 0.348), and age of patients (*p* = 0.768) (Table 3).

**Table 2:** EH grade in vestibule and lateral semicircular canal.

	**Vestibule**	**Lateral semicircular canal**
None	8	29
Grade I	14	23
Grade II	22	8
Grade III	21	5

**Table 3:** Results of vHIT and Caloric test in patients.

	**CP (+)**	**CP (–)**	**Total**
VOR gian (+)	13	1	14
VOR gain (–)	25	26	51
Total	38	27	65

*Chi-square test, correction for continuity. Statistically significant. χ^2^ = 6.981, p = 0.005.*

*CP, canal paresis; VOR gain, vestibulo-ocular reflex gain*.

### Correlation Between Vestibular Function Tests and MRI

#### VEMP

**Figure 3:**
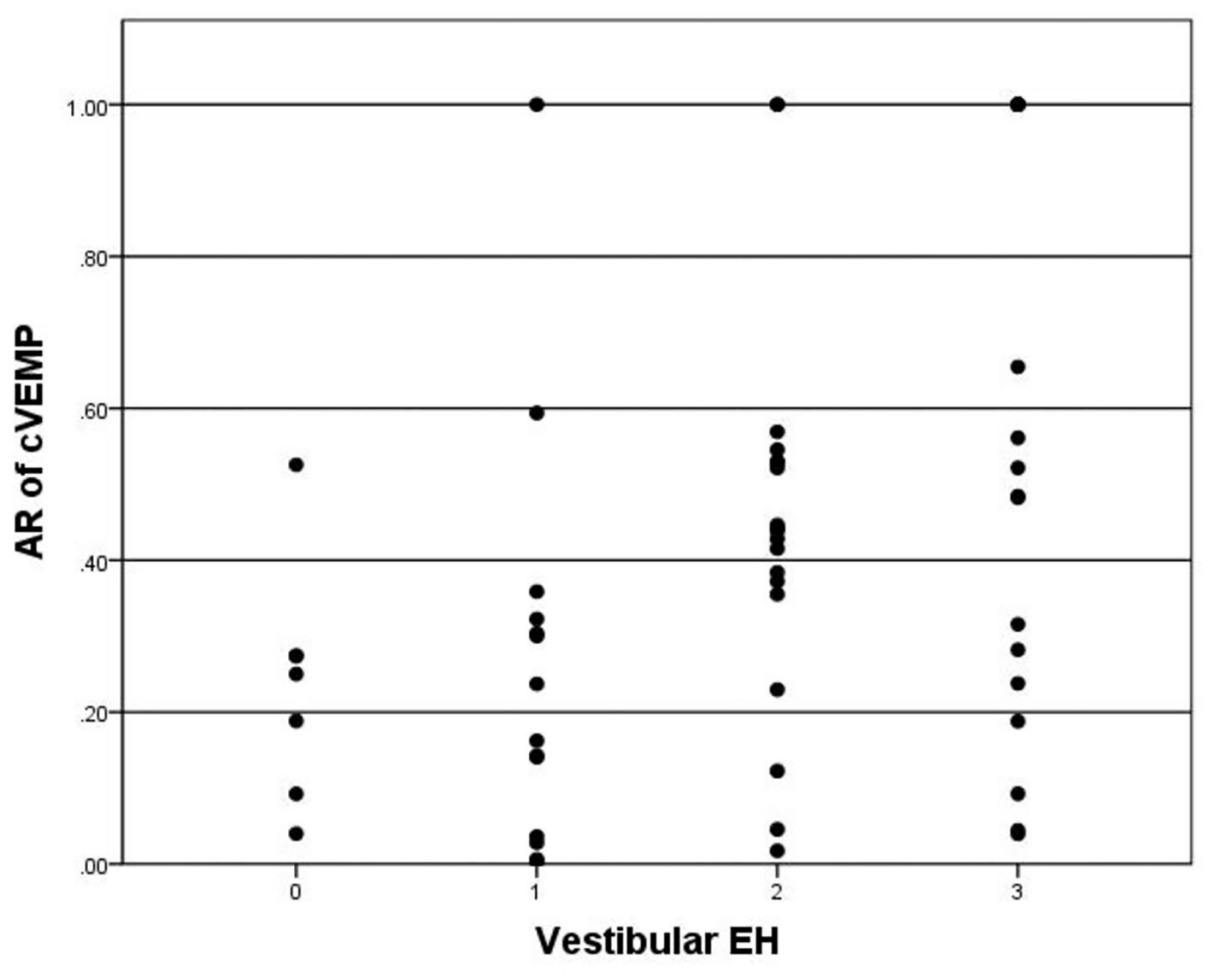
Correlation between grade of vestibular EH (x-axis) and AR of cVEMP (y-axis). The positive correlation is significant (*r* = 0.407, *p* = 0.001).

A total of 6 patients showed bilateral absence of cVEMP were excluded in the correlation analysis with hydrops on MRI. cVEMP was elicited in 48 of 65 (73.8%) patients. The P13–N23 amplitude of the affected side was significantly smaller than the contralateral side (*p* < 0.05). The mean AR of cVEMP was 0.42 ± 0.32. The Spearman correlation analysis revealed no correlations between vestibular hydrops and the latency of P13 (*p* = 0.390), the latency of N23 (*p* = 0.056), or the amplitude of P13–N23 (*p* = 0.109). Vestibular hydrops is significantly correlated with AR of cVEMP (*r* = 0.407, *p* = 0.001) (Figure 3).

**Figure 4:**
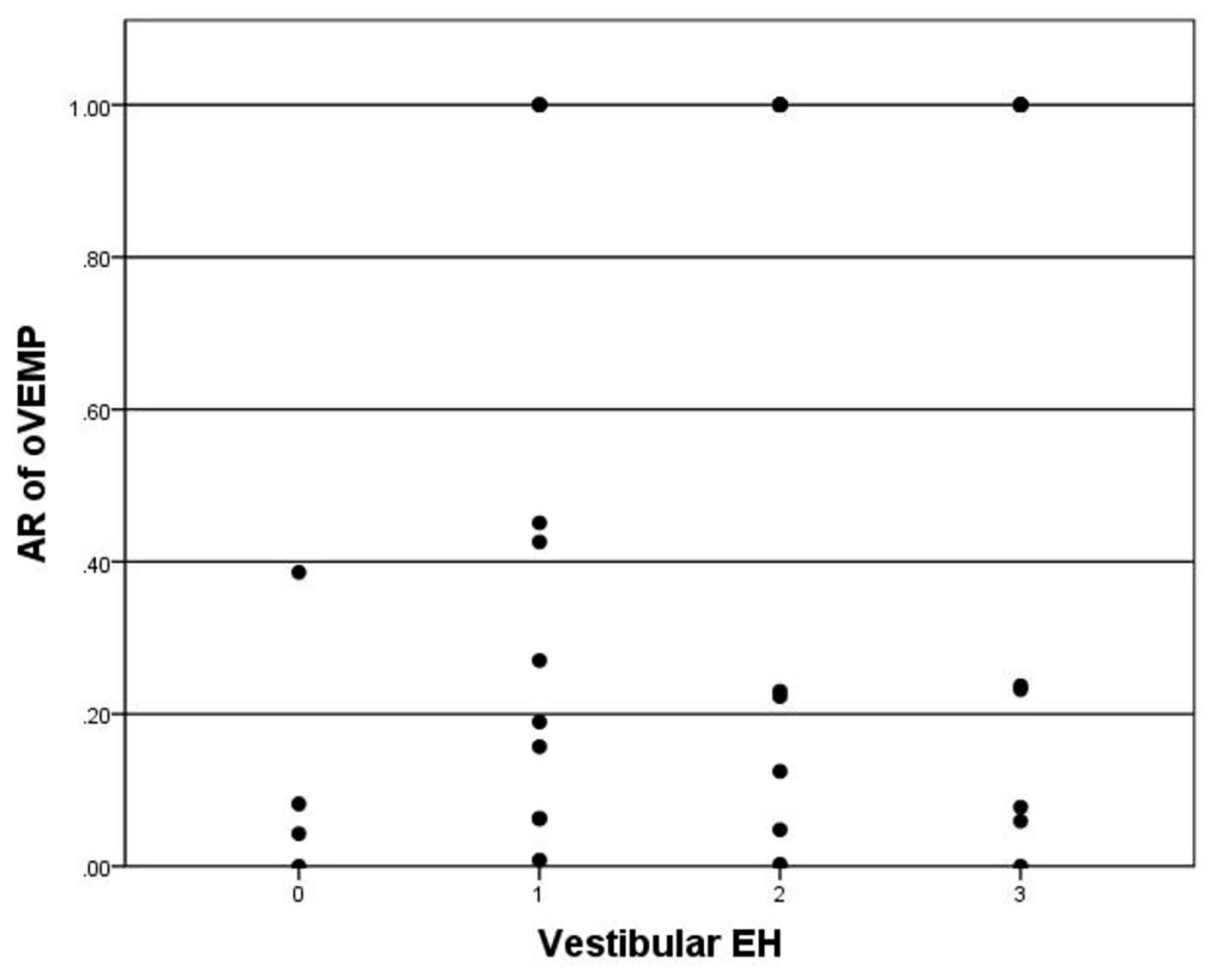
Correlation between grade of vestibular EH (x-axis) and AR of oVEMP (y-axis). The positive correlation is insignificant (*p* = 0.098).

For oVEMP, a total of 28 patients showed bilateral absence of oVEMP were excluded in the correlation analysis with hydrops on MRI. oVEMP was elicited in 26 of 65 (40.0%) patients. The P13–N23 amplitude of the affected side was significantly smaller than the contralateral side (*p* < 0.05). The mean AR of oVEMP was 0.49 ± 0.43. Vestibular hydrops showed no significance in correlation with AR of oVEMP (*p* = 0.098) (Figure 4). Also, there were no significant correlations between vestibular hydrops and the latency of P13 (*p* = 0.976), the latency of N23 (*p* = 0.590), the amplitude of P13–N23 (*p* = 0.090).

#### Caloric Test and vHIT

**Figure 5:**
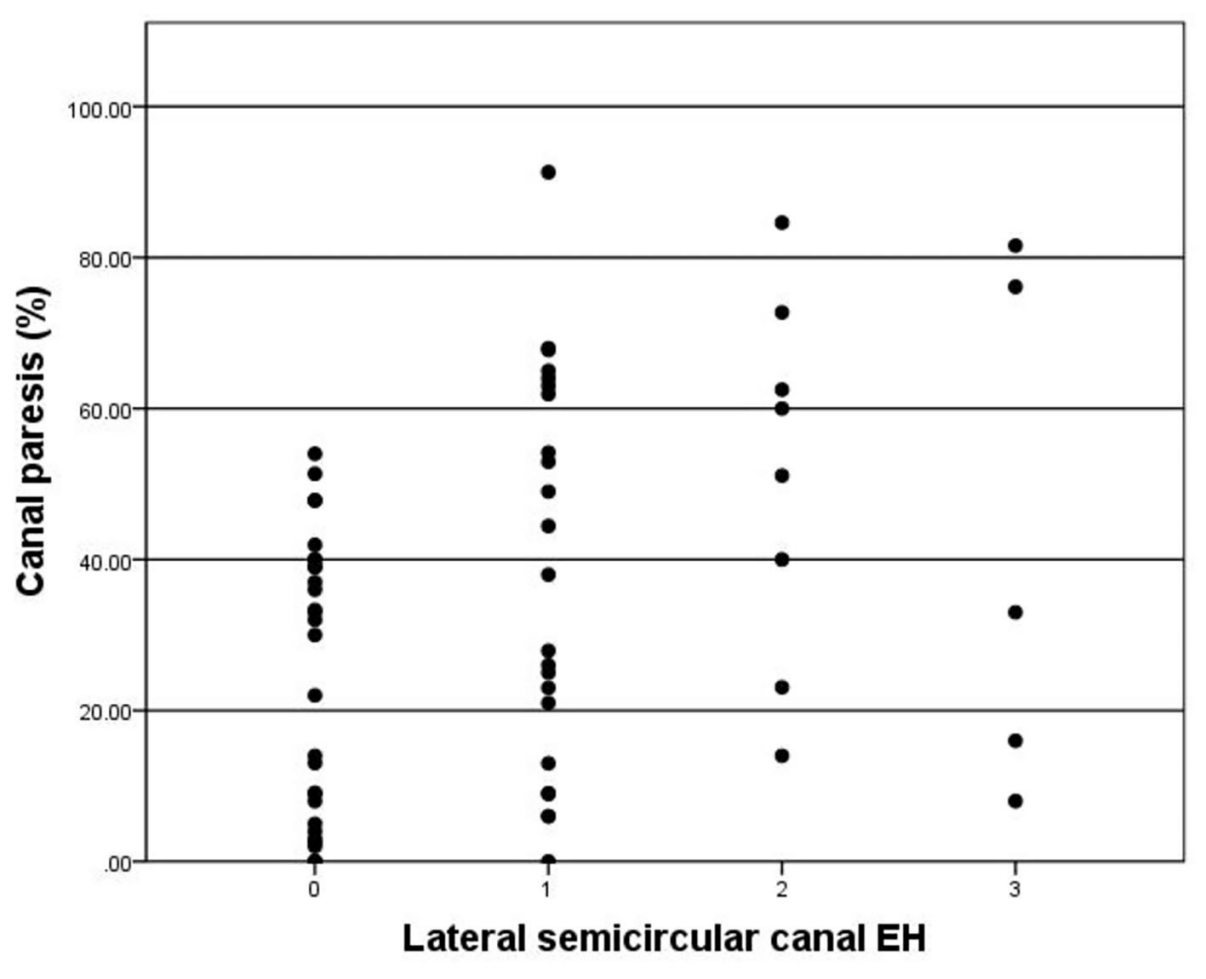
Correlation between grade of lateral semicircular canal EH (x-axis) and CP (y-axis). The positive correlation is significant (*r* = 0.367, *p* = 0.003).

CP was observed in 38 of 65 (58.5%) patients. CP was significantly correlated with lateral semicircular canal hydrops (*r* = 0.367, *p* < 0.003) (Figure 5). No correlation was observed between CP and vestibular hydrops (*p* = 0.365).

**Figure 6:**
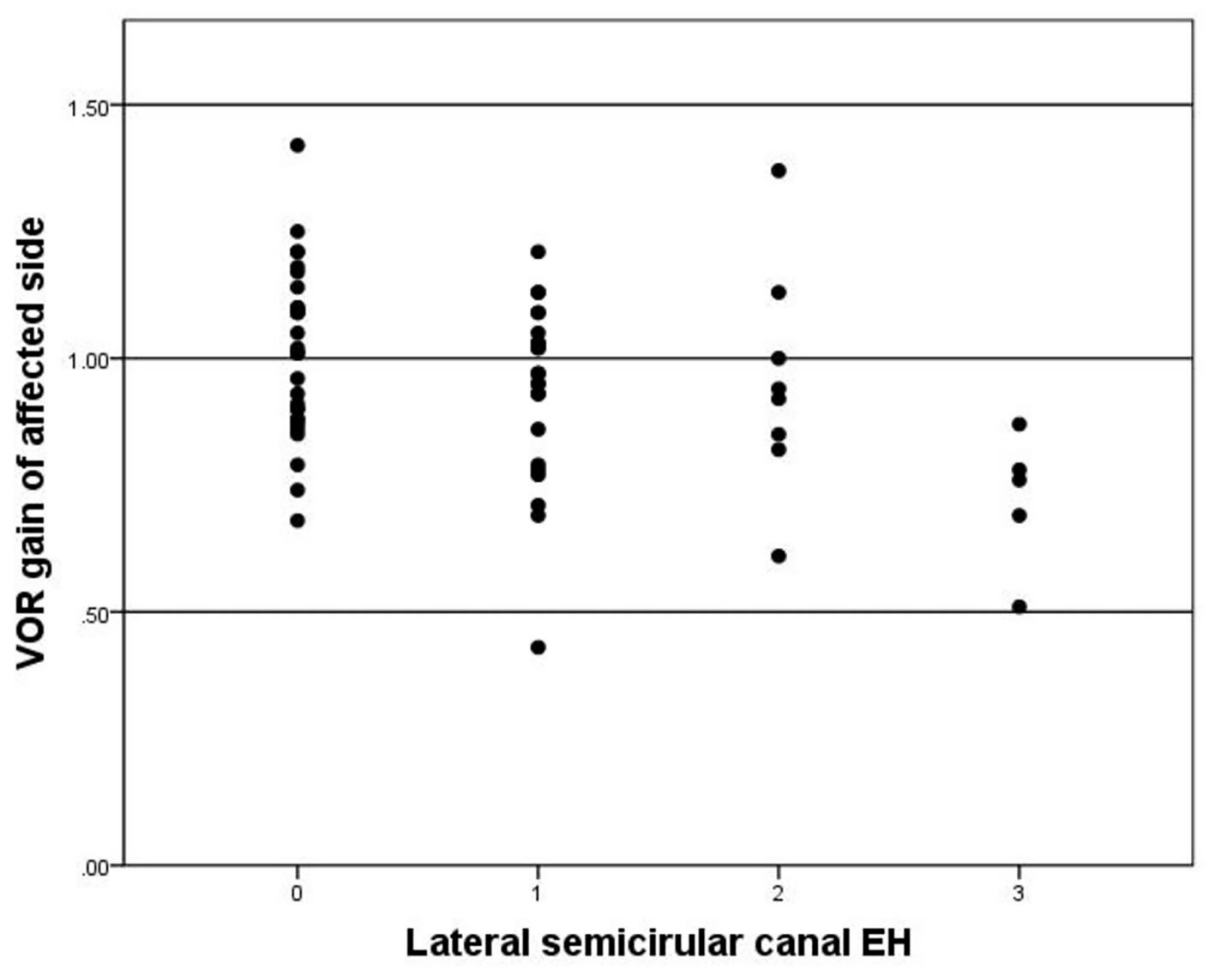
Correlation between grade of lateral semicircular EH (x-axis) and VOR gain of affected side (y-axis). The negative correlation is significant (*r* = −0.311, *p* = 0.012).

Abnormal VOR gain was observed in 14 of 65 (21.5%) patients. The mean VOR gain on the affected side was 0.95 ± 0.19 and 1.11 ± 0.15 on the contralateral side. The VOR gain of affected side is significantly lower than the contralateral side (*p* < 0.05). VOR gain was significantly correlated with lateral semicircular canal hydrops (*r* = 0.311, *p* = 0.012) (Figure 6). There was no significant correlations between vestibular hydrops and VOR gains (*p* = 0.243).

## Discussion

The pathological change of MD is characterized by EH. Clinical diagnosis of MD is mainly based on the combination of medical history and auditory-vestibular function examination. With the development of Gd-contrasted inner ear MRI, clinicians are now able to observe this most important pathologic feature of MD by *in vivo* MRI. According to the previous studies, the accuracy for clinical diagnosed MD is 56–73% for electrocochleogram, VEMP, and caloric test (20–22). On the other hand, Gd MRI demonstrated EH in 90% of the patients with MD in a multicenter clinical study (23). The *in vivo* visualization of EH has become a powerful tool for the investigation of MD. Furthermore, the significance of EH and vestibular function tests in patients with MD has not been fully characterized yet. The recent research published by our team modified the grading system of vestibular and semicircular canal hydrops (15). The “volume-referencing” grading system showed a better correlation with the MD clinical features and had more diagnostic value than previously published methods. But the correlation between this grading system and vestibular function tests requires further research.

In this study, four patients (4/69, 14.5%) were excluded in the final data analysis because no signs of any contrast medium could be observed in any part of the endolymph. We considered the possible cause of this phenomenon is that the permeability of the round window membrane is poor and the concentration of the contrast agent into the inner ear is insufficient. Yoshioka et al. (24) evaluated the permeability of the round window membrane in 42 MD patients and 13 non-hydrop inner ear disease patients by 3D-FLAIR MRI after intratympanic Gd injection. The results showed that 13% of the patients had poor permeability of the round window membrane, and nearly half of them had calcified plaques n the tympanic membrane or inflammation in the mastoid. This proportion is similar to that of our study. The permeability of the round window membrane is an unpredictable reason for the failure of inner ear MRI after intratympanic Gd injection.

The relationship between the degree of EH and clinical features, such as duration of the disease and hearing loss was previously reported by researchers. In this study, we confirmed that vestibular EH was correlated with duration of disease and PTA. Gurkov et al. (25) reported that Patients with a longer disease duration had a higher degree of EH. A significant positive correlation between hearing loss and cochlear EH was also confirmed. But the correlation between vestibular hydrops and hearing loss or duration of disease was not mentioned. Wu et al. (26) revealed that there was a significant correlation between the duration and vestibular EH. PTA thresholds at low and middle frequencies were significantly correlated with the extent of EH in cochlea. No significant correlation was detected with the vestibular hydrops and PTA. Yang et al. (27) demonstrated both vestibular and cochlear EH were significantly correlated with PTA. But they concluded that duration of disease was not correlated with vestibular EH or cochlear EH. Fiorino et al. (28) concluded that the severity of vestibular EH is positively correlated with the course of disease, especially in patients with more than 5 years of disease duration. The results are consistent with the pathological findings of the temporal bone (29). The above results suggest that the EH in patients with MD is progressive aggravation, However, the mechanism of this phenomenon in MD remains to be further studied. The ideal method to study the correlation between the duration of disease and EH should be the large scale longitudinal study in different course of disease in the same patient. But its feasibility is limited due to the economics and ethics considerations.

VEMP has been gradually widely applied in the clinical diagnosis and research in various kinds of peripheral vestibular diseases. cVEMP can reflect the function of saccule and the integrity of its pathway. The pathway is from the saccule, inferior vestibular nerve, vestibular nucleus, accessory nucleus, accessory nerve to sternocleidomastoid muscle. oVEMP can reflect the function of utricle and the integrity of its pathway. The pathway is from the utricle, superior vestibular nerve, vestibular nucleus, contralateral medial longitudinal fasciculus, contralateral oculomotor nucleus to contralateral inferior oblique muscle (2). The elicited response rate of cVEMP and oVEMP was 73.8 and 40.0% in this study. The response rate of oVEMP was lower than the data previously reported (30, 31). The possible reason is that 78.2% (55 of 69) patients included in this study were in stage III and stage IV. The response rate and abnormal rate of cVEMP and oVEMP in MD increased with the progression of the disease and the severity of hearing loss (32). The latency both in cVEMP and oVEMP showed no correlations with vestibular EH in our study. Researches have revealed that the latency of VEMP is prolonged due to the damage of the vestibular nerve pathway in the retrolabyrinthine (33). However, different conclusions have been made in the researches of MD patients. Johnson et al. (34) revealed that the latency of P13 was prolonged in the affected side of MD patients when compared to the contralateral side and the healthy controls. On the other hand, Murofushi et al. (35) patients with MD or vestibular neuritis hardly showed any latency prolongation in cVEMP. Whether the vestibular nerve pathway in the retrolabyrinthine of MD patients is damaged still need to be clarified in the future study. Since the amplitude of VEMP can be affected by muscle tension and subcutaneous thickness, Young et al. (36) put forward the concept of AR, and considered that AR is a more accurate method to evaluate the vestibular function of both sides, which can reflect the symmetry of vestibular function in MD patients. The present study demonstrated that AR of cVEMP was correlated with vestibular EH while AR of oVEMP was not. Gurkov et al. (25) drew a similar conclusion with our study, which indicated that patients with a more severe degree of vestibular EH also had a worse sacculus function. Guo et al. (6) have also reported that cVEMP AR was higher in MD patients with severe vestibular hydrops than in patients without vestibular hydrops. The phenomenon matches the theory that severe vestibular EH would lead to permanent morphological changes of the sensory organs, including the loss of saccular macula associated with the collapse of the saccular wall onto the otolithic membrane, which is consistent with reduced cVEMP (29). Quantitative analyses of the relationship with oVEMP values and EH degrees in vestibule in MD patients are rare. Jerin et al. (37) concluded that the 500/1,000 Hz amplitude ratio was neither correlated with the degree of endolymphatic hydrops nor with the duration of disease. The correlation between oVEMP and EH need to be further studied.

In the present study, CP was observed in 38 of 65 (58.5%) patients while abnormal VOR gain was only observed in 14 of 65 (21.5%) patients. The function of semicircular canal has frequency specificity. The caloric test was a function test at low frequency, while the VHT was a test of the high frequency. They are complementary. The abnormal rate of caloric test in patients with MD is higher than that of vHIT. The probable explanation is that the low frequency function of the semicircular canal decreased firstly, and then the high frequency function impairment occurred later in MD patients, because the caloric test examines the response of the semicircular canal at a low frequency and non-physiological stimulation, which the daily activities can not compensate for its functional decline. While the vHIT examines the high frequency function of the semicircular canal, and the frequency of head position movement stimulation in daily activities is relatively consistent with the high frequency. Vestibular compensation plays a vital role in the high frequency function impairment in patients with MD. Thus, high frequency function impairment occurs later in the duration of the disease and maintains a relative balance between the two sides (38). There were few reports of the semicircular function related to EH detected with MRI. In previous studies, Kahn et al. (8) reported no significant correlation between ampullar hydrops and vHIT by a simple two-grade canal hydrops evaluation. Gurkov et al. (25) demonstrated no significant correlation between the severity of vestibular EH and CP. Fukushima et al. (39) revealed that vestibular EH was not correlated with VOR gains, but correlated with CP. However, both CP and VOR gains of lateral semicircular canal showed significant correlations with hydrops according to the modified canal hydrops grading system proposed by our team. Although a single vHIT or caloric test has limited sensitivity to MD, especially in the early stage of the disease. But as the disease progressing, changes of CP and VOR gains of lateral semicircular canal were able to represent the degree of lateral semicircular canal hydrops to a certain extent.

These limitations should be taken into consideration is this study. First, the results of vestibular function test were short of normal healthy population in local area as reference. Adapting the standard values reported by the previous literature in the study would lead to result error. Second, the reliability of the statistical analyses would profit from a greater number of patients. Third, in this study, 78.2% of the patients were in stage III and stage IV, and most of them had significant vestibular hydrops. In some cases, it was difficult to evaluate the degree of EH in the lateral semicircular canal because of the close anatomic connection between the utricle and the ampulla of the lateral semicircular canal. The number of patients with no or mild EH was relatively small. Thus, for more precise study between canal hydrops and caloric test and vHIT, a study including more patients of stage I and stage II is necessary.

## Data Availability Statement

The raw data supporting the conclusions of this article will be made available by the authors, without undue reservation.

## Ethics Statement

The studies involving human participants were reviewed and approved by The Ethics Committee of Xinhua Hospital, Shanghai Jiaotong University School of Medicine. The patients/participants provided their written informed consent to participate in this study.

## Author Contributions

JY and MD contributed to the study design, critically reviewed, and approved the final manuscript. YL and FZ contributed to the detailed study design and performed data acquisition, statistical analysis, and interpretation of results, drafting of the manuscript, and revised the manuscript. BH, JH, and QZ contributed to the methods of statistical analysis and critically reviewed the manuscript. All authors agree to be accountable for the content of the work, integrity, accuracy of the data, contributed to the article, and approved the submitted version.

## Conflict of Interest

The authors declare that the research was conducted in the absence of any commercial or financial relationships that could be construed as a potential conflict of interest.
